# Quitting the quitline: a qualitative study of patient experience of electronic referrals to quitlines

**DOI:** 10.1186/s12889-020-09185-4

**Published:** 2020-07-09

**Authors:** Elizabeth L. Albert, Jeanmarie C. Rose, India J. Gill, Susan A. Flocke

**Affiliations:** 1 Center for Community Health Integration, Case Western Reserve University, 10900 Euclid Avenue, Cleveland, OH 44106-7136 USA; 2 Department of Family Medicine, Oregon Health & Science University, Portland, OR 97239 USA

**Keywords:** Tobacco quitlines, Tobacco cessation, Program use, Primary care, Qualitative research

## Abstract

**Background:**

The use of electronic referrals (eReferrals) to state quitlines (QLs) for tobacco-using patients is a promising approach for addressing smoking cessation on a large scale. However, QL contact, enrollment, and completion rates are low. The purpose of this study was to examine the eReferral to QL process from the patient’s perspective in order to inform strategies for improving QL engagement.

**Methods:**

We conducted interviews with 55 patients who agreed to an eReferral at a primary care visit to 1 of 8 safety-net community health centers in Cuyahoga County, Ohio (September 2017–August 2018). Interviews were designed to explore the experiences of three subgroups of patients who subsequently: 1) declined participation in the QL; 2) were unreachable by the QL; or 3) were enrolled in or had completed the QL program. Analysis was guided by a phenomenological approach designed to identify emergent themes.

**Results:**

Reasons for QL program non-completion included changing life circumstances and events making cessation unviable; misunderstandings about the QL; discomfort with telephonic counseling; perceived lack of time for counseling; cell phone barriers; and having already quit smoking. We found that some individuals who were no longer engaged with the QL still desired continued support from the QL.

**Conclusions:**

Participants intentionally and unintentionally disengage from the QL for a wide variety of reasons, several of which are mediated by low socioeconomic status. Integrating QL care with community-based resources that address these mediators could be a promising strategy.

## Background

Tobacco smoking accounts for about 1 in 5 deaths in the United States [1], and is more prevalent among those who live below the poverty level [2], making smoking cessation a public health priority [3]. Although 68% of adult smokers report wanting to quit, less than one third of those used evidence-based cessation methods when trying to quit [4]. Quitlines (QLs) have become a key public health strategy for delivering evidence-based, cost-efficient tobacco cessation assistance to those interested in quitting smoking [5–7]. While overall quit rates in the US are 7.4% [4], quit rates for those who use a QL are 30.3% [8].

Referral to QLs by health care providers is strongly recommended as an effective strategy for providing tobacco cessation assistance [9–11], and dissemination of QL-delivered treatment within the context of a health system has the potential to address smoking cessation on a large scale. In particular, systems changes in which providers electronically submit referrals to the QL and the QL proactively contacts patients, a process known as eReferral, have been shown to increase the proportion of tobacco users referred by 3–4-fold [12], and the proportion who receive treatment from a QL by 13-fold [13].

There is growing evidence, however, that with this approach, the QL is unable to contact and/or keep enrolled a large percentage of the patients who agree to be connected [12, 14–16]. In a study evaluating an e-Referral system linking patients visiting two healthcare clinics in a regional health system (one primary care, one pulmonary medicine) to the state tobacco QL, Adsit et al. found that, although there was an increase in the number of referrals to the QL, among those consenting to be eReferred, 64.8% later declined QL cessation services [14]. Bui et al. examined differences in QL enrollment among smokers seeking care at 1 HIV clinic and 12 non-HIV clinics that were part of a large healthcare system in a large metropolitan area. They found that at the non-HIV clinics, 54.5% of referred smokers were later unreachable, and of those reached, 24% then declined services; similarly, among those seen at the HIV clinic, 55.6% of referred patients were unreachable and 24.9% of those reached declined services [15]. In addition to being unreachable or declining QL participation, many smokers do not complete the multiple (3-5) counseling sessions offered by most QLs [17, 18]. This is significant because research has found that those who complete more of the calls offered in QL programs have higher quit rates than those who complete fewer calls [7, 18, 19].

Two studies have examined characteristics associated with completion of QL programming. Burns et al. [20] found that factors that predicted completion of only one session (vs. more than one session) included not being sent nicotine replacement therapy (NRT); being 18–24 years old, female, or African American; having a high school education or less; having no insurance, previous quit attempts, or any children in the home; smoking < 20 cigarettes per day, and living with a tobacco user. However, none of these factors accounted for more than 2% of variance. Lien et al. [17] assessed whether intensity of QL use was associated with participant characteristics in Minnesota and Pennsylvania. Results were similar in each state, with few (8–11%) completing all five calls and many (37–46%) completing only one call. Greater intensity of QL use was associated with older age and having chronic or mental health conditions. While important, there is a need for additional research to better explain why many people do not fully participate in QL programming.

No studies have engaged eReferred smokers to explore their experience of the eReferral-to-quitline process, nor have any studies attempted to reach those who were unable to be reached by the QL or declined QL participation. The current study addresses this need by examining patient experience from the point of the offer of the eReferral by a health care provider to the conclusion of contact with the QL provider. The objective of this study is to understand the barriers to the completion of the QL protocol, and to identify ways to improve patient engagement with the QL.

## Methods

### Overall study frame

This study was part of a larger project to implement a systems-based intervention that establishes a proactive eReferral capacity to the Ohio Quitline. The intervention includes an Ask-Advise-Connect strategy to electronically refer interested patients to the QL, prompting a proactive call to the patient to invite them to enroll in counseling sessions [13, 21]. We partnered with MetroHealth, a safety-net health system in Cuyahoga County, which includes Cleveland, Ohio, and serves the largest portion of Medicaid and uninsured patients in the region. In addition, 26% of MetroHealth adult primary care patients report using tobacco, compared to the national estimates of 13.7% [22]. The intervention was implemented in 8 community health centers in the MetroHealth system. Among patient visits to these sites during 2017–2018, 70% were female, 50% were white and 46% were black, 40% had Medicaid, 24% Medicare and 6% were uninsured.

### eReferral to quitline process

The eReferral process, called Ask-Advise-Connect, was activated at the visits of all patients seeing their primary care provider for a routine visit. The medical assistant (MA), the individual who rooms the patient and completes the intake process, begins by **A**sking the patient their current smoking status. If the patient affirms current smoking, the MA was instructed to **A**dvise the patient to quit using tobacco and assess the patient’s interest in quitting in the next 30 days. Patients interested in quitting and in receiving assistance from a smoking cessation counselor were **C**onnected to the Ohio Quitline by the MA placing an electronic referral via the EHR.

**Fig. 1:**
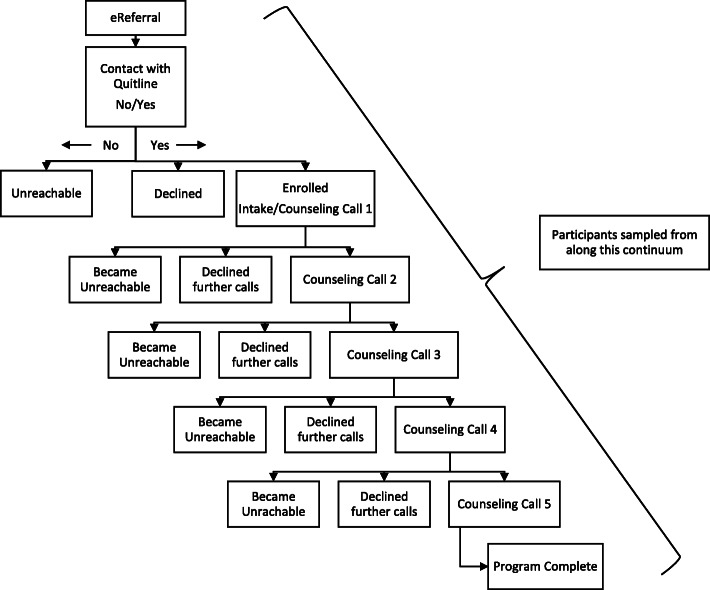
EReferral-to-Quitline Continuum

Once an eReferral was sent to the QL, QL counselors attempted to call the participant within 24 h (See Fig. 1). If after 5 call attempts no contact was made, that participant was considered ‘unreachable’. If contact was made, the QL counselor would briefly describe the program and the participant could choose to ‘decline’ or ‘enroll’ in the program. Upon enrollment, the QL counselor would administer a short intake process focused on tobacco use history and the participant could begin receiving counseling on that first call. The QL provides up to five counseling session calls, making five call attempts for each session, and leaves a message if the patient does not answer the phone. As shown in Fig. 1, once enrolled, participants can choose to dis-enroll either by formally declining further participation or becoming unreachable, and may do so after one or more counseling sessions. Participants who complete all five counseling calls are considered to have completed the program.

### Sampling

Monthly data was requested from the QL and included variables pertaining to the patients’ progress through the QL program, such as current QL enrollment status (not enrolled, enrolled, dis-enrolled) and reason (unreachable, declined, program complete), and number of coaching calls. We used purposive sampling to select participants categorized as unreachable, declined and enrolled, and who were in varying stages along the QL continuum, including ‘program complete’ (See Fig. 1). With this iterative approach, as analysis of the initial cases progressed, additional cases were then selected in order to fill in the gaps of understanding.

### Recruitment

Patients selected for recruitment were first sent an email or postal letter notifying them that they may be eligible to participate in a study and that a study team member would contact them by phone. The study team made two call attempts to reach participants to invite them to participate. If there was no answer, a brief message with a return number was left and the study team attempted calling participants back at their preferred time.

### Data collection

Patients who agreed to participate were interviewed by one of two study team members, both trained in conducting in-depth interviews. First, the consent script was read by a team member verbatim to the patient before the interview began. As the interviews were conducted by phone, all patients provided verbal consent to participate. The Institutional Review Board of MetroHealth approved the use of verbal consent to participate. The interviews were conducted using a semi-structured interview guide that was developed for this study and is included as an additional file [See Additional file 1]. The interview guide was designed to elicit participants’ thoughts on their smoking habits, quitting smoking, discussing smoking with their primary care providers, their eReferral experience, and their QL program participation. After several interviews, the interview guide was modified to include additional probing questions to better understand what was most helpful in their interactions with the QL, and why some participants remained unreachable or declined engagement with the QL. Additional questions about the participants’ past history of smoking were also added to provide more historical context to participants’ current progress or barriers with quitting. Interviews lasted between 9 and 34 min, with an average of 18 min, and were audio-recorded. Participants were compensated with a $25 gift card. Data collection took place between September 2017 and August 2018. All study procedures were approved by the Institutional Review Board of MetroHealth.

### Analysis approach

All interviews were transcribed verbatim. Phenomenology is an approach to understand how people make meaning of their lived experience [23]. This approach helps researchers explore what is being experienced, and also how it is experienced by the participants. We applied this approach to develop a deeper understanding about the common features shared among individuals who agreed to the QL connection and to understand their experiences with the referral process with the MA, the QL calls, and the QL coach. Analysis began with careful and repeated reading of several transcripts by three trained analysts to identify salient themes of the QL referral process. Based on this initial round of thematic analysis, an initial set of coding categories was created. As additional transcripts were read, the coding categories were modified as necessary to better fit the themes that emerged. Next, two of the analysts independently coded all 55 transcripts, meeting regularly to discuss coding, and reach consensus on any discrepancies. Patterns across codes were described and supported with example quotes. These emergent themes were reviewed and discussed with a third analyst to ensure representation and clarity. Additional interviews were conducted until the point of data saturation was reached for each of the categories: declined, unreachable, enrolled, program complete.

## Results

The characteristics of the 55 individuals that agreed to be interviewed versus those that were invited but did not participate in the interviews are displayed in Table 1. Our iterative sampling required us to contact 170 individuals to achieve the sample of 55 (31% participation rate). Participants were an average age of 50.6 years; 76% were female, 58% were African American, and 57% received Medicaid. Overall, those that participated were similar across characteristics to those that did not participate.

**Table 1:** Characteristics of interview participants and non-participants

Characteristic	Total *N* = 170	Participants *n* = 55	Non-participants *n* = 115
Age, mean (std dev)	51.3 (13.2)	50.6 (12.0)	51.6 (13.8)
Female	108 (64%)	42 (76%)	66 (57%)
African American	95 (56%)	32 (58%)	63 (55%)
**Insurance type**			
Commercial	4 (2%)	1 (2%)	3 (3%)
Medicaid	106 (62%)	31 (57%)	75 (65%)
Medicare	58 (34%)	21 (39%)	37 (32%)
Self-pay	1 (1%)	1 (2%)	0 (0%)

Our analysis generated six themes, or clusters of meaning, that shed light on the common experiences, motivations and understandings of those who did not complete the available five counseling sessions offered by the QL. We found that these themes crosscut the QL categories of ‘declined’ and ‘unable to be reached’. These themes are not mutually exclusive, and many patients reported more than one theme. We discuss each of the themes below. Additional example quotations for each theme are provided in Table 2.

**Table 2:** Thematic quotations from interviews with smokers e-referred to the Ohio Quitline

**Differing expectations regarding the quitline referral** “You know, and I just wasn’t sure (what to expect). To be honest with you, I thought it was a program like a friend of mine went through some years ago where he actually went like to one of the clinics in the evenings.” [ID #08] [Describing a visit during which the patient was in too much pain to ask questions clarifying the QL referral] “The only thing I got was ‘You call them, and they’ll call you back.’ That’s the only thing I got … I wanted to ask, but I was in pain … I wanted to know about like if you go to the hospital and you sit down with somebody. I wanted to ask those questions, but I honestly was not feeling good and I didn’t wanna hear anything.” [ID #14] “I wanted patches. So that’s what I thought I was gon’ get, some patches. Like I was saying, that’s not what I wanted to do - them checking on me and this and that and all that … So you know, I told them, ‘That’s all right.’ If I couldn’t get the patches, that’s all right. That was the end of that conversation. [ID #25].” **Changing life circumstances and stressors** “My mother had a stroke, and but she wasn’t doing too good and wasn’t nobody here to take care of her, but me. You know how hard that was on me. She was bedridden, and she died like in August of last year. Yeah, and trying to take care of myself and trying to take care of her. It was a lot.” “I just had a lot going on. A lot of issues with family, things going on lately. Our home was just burglarized a couple weeks ago. I’ve just had a lot going on … I mean it’s definitely something I’d like to do eventually. It’s just gotta be the right time, and a lot of times when you try to quit something and then you go back to it, becomes, you know it just becomes worse, I guess.” [ID #33] “Well they sent me the brochures and everything. But my mindset wasn’t in the right frame of mind at that point, ‘cause my dad was in a nursing home. And he just recently passed away, so I wasn’t really in the right state of mind back then. It was kind of stressful, and that was like the stress-relief to get out the nursing home and have me a cigarette and go home.” [ID #44] **Unable to find time for counseling** “I’ve been at work so much that I never get a chance to conversate with them, ‘cause I’m at work like from morning ‘til late evening.” [ID #13] “I actually received a couple calls that I missed because I was at appointments, or I was either at my kids’ school or something and didn’t answer the phone.” [ID #12] “Usually when they call, sometimes I don’t answer because I’m either picking up my kid, or taking him to school … and then with Christmas, holidays. Everything is just, you know, and then trying to figure out with the doctor ‘cause well I just had another episode, so I was in the hospital.” [ID #22] **Cell phone barriers** “They probably tried to call me, but my phone’s been stupid … I cracked it, so sometimes it answers and sometimes it doesn’t.” [ID #54] “Yeah, and then the phone I had, I lost it, and I ain’t been able to afford me another phone, but I got a birthday soon coming. I guess they’ll pitch in and buy me a phone, and I’m using a temporary phone now until I get me another real good phone. I had phones and kept having problems with them.” [ID #52] “The MA asked if I had any interest in stopping and I told her yes, and then she gave me or told me about the quitline was supposed to call me, which I believe they may have, but the number comes up and if it’s an 800 number, I usually don’t answer it because you know it doesn’t come up under the quitline, you know, ID. It just comes up as an 800 number.” [ID #04] **Discomfort with/disbelief in the efficacy of quitline counseling** “I did (agree to be connected to the quitline), and we did speak. Someone did call me with the department of the quitline, and I was not comfortable. I’m not gonna lie to you … I think it was just the person that spoke to me over the phone. In reality, I know that it’s just your job to try to give information out, or try to help someone, but you need to feel comfortable with somebody when you speak to them over the phone, and I just didn’t feel comfortable with the first call I got. So I didn’t agree to the over-the-phone line quitting situation because, I don’t know. I just didn’t feel comfortable.” [ID #15] “You know the first time, the lady was professional and generous. It’s just I don’t think it was very helpful to me.” [ID #41] “Cause talking with somebody about quitting doesn’t do any good. I feel like talking wouldn’t do any good. ‘Cause I would go ahead on and say ‘Yeah. Um hmm. Yeah. You’re right. You’re right.’ and it’ll be going in one ear, coming out the other.” [ID #23] **Quitting on their own** “I got a call and they asked me, you know they said ‘Are you interested in quitting?’ and I said ‘Yeah.’ I told them I was in the process of trying to quit then, you know, and they told me if I needed help, to get in touch with them.” [ID #28] “They called me, but I didn’t really speak with them because I actually stopped, and I didn’t need the help. And I’ve been doing good ever since then.” [ID #03]	

### Reasons for QL disengagement

#### Differing expectations regarding the QL referral

The degree to which participants understood what the QL counseling process involved varied a great deal. In some cases, participants expected the cessation counseling to be in-person, at a local hospital or clinic. When these participants became aware that the counseling was via telephone, some were uncomfortable with the prospect of speaking with a “stranger” or “random people”. In other cases, individuals did not understand they were accepting a referral. At the time, they were experiencing symptoms related to the reason for their visit to the doctor, such as trouble breathing, or intense pain: “I’m in too much pain sometimes when I go to the doctor. So I’m just like ‘Okay. Yeah, I know. I know.’” [ID# 14] Another area of misunderstanding was the role of the QL in supporting smoking cessation. Some participants believed the QL simply provides NRT free of cost, and had no interest in counseling: “Basically they was telling me that you gotta do counseling and stuff, and that’s not what I was looking to do. I was basically looking just to get the patches and try to do it on my own.” [ID# 39].

#### Changing life circumstances and stressors

Another pervasive theme was that stress from traumatic events or changing life circumstances made it difficult for many participants to begin or stick with the program, and in some cases resulted in the participant no longer feeling ready to quit. The circumstances mentioned by these participants included stressors such as housing instability, serious illness or hospitalization, recent death of a loved one, and being the victim of a crime. One individual, who was living in a transitional housing unit, reports: “They call me every two weeks to coach me to stop smoking. But like I told them this past week - I was being honest with them ‘cause I was avoiding their calls when they came through - I never did stop smoking, or slow down, due to the fact that I am stressed big time, living in a box … And it’s a whole lot of other stressful stuff going on in my family, of me burying people. And I’m just out of it. So I’m not really on the right track of stop smoking.” [ID #24].

#### Unable to find a time for counseling

Several participants expressed that, although they were interested in quitting smoking, they found it difficult to find time to engage in a QL counseling call. Participants spoke of having a job or multiple jobs that kept them too busy to contact the QL, or unpredictable work schedules that made it difficult to keep appointments with the QL. In addition, many participants had responsibilities such as taking care of children, parents and other family members. These priorities often meant that QL counseling was either intentionally or unintentionally put on the backburner. Engaging in counseling sessions with the QL “takes too much time out of my days, and I don’t have the time”. [ID #39].

#### Cell phone barriers

Access to a properly functioning phone with a consistent number and uninterrupted service was a common barrier for the QL reaching participants. For some, a disruption in cell service precluded the QL from making contact with a participant: “I know that [the MA] recommended me, and the people were supposed to call me from the 1-800-quitline. But the time they was calling, my phone was cut off.” [ID #19] Further, if participants reported that their cell phone was lost, stolen, or no longer functioning, replacing it was not always viable immediately. In some cases, they had to wait until funds were available. Additionally, the QL phone number is a 1–800 number that does not appear with a description in caller ID. Some individuals were suspicious of unidentified numbers and/or had a policy of not answering 1–800 numbers. Although the QL does leave messages, many participants reported not listening to their messages, especially if it was from an unknown number.

#### Discomfort with/disbelief in the efficacy of QL counseling

Another reason for declining the QL program or being unreachable was discomfort with the phone counseling experience, or the belief that it would not help them quit. Some participants did not feel a personal connection to the counselor, and therefore felt hesitant about discussing their situations. Others, even while acknowledging that the QL information was helpful, did not feel the counseling process would actually help them quit smoking: “I never knew about the phone therapy. That was my first time experiencing it, knowing about it. So I tried it out and I didn’t like it.” [ID #24].

#### Quitting on their own

A few unreachable or disenrolled participants, as identified by the QL, had already quit smoking or cut back significantly prior to the completion of the QL protocol. These individuals felt confident in their ability to stay tobacco-free or continue the quitting process, and felt they no longer needed assistance from the QL.

### Quitline benefits and ongoing support

Another common theme that emerged among those who had received any QL counseling, including those who at some point disengaged and did not complete the protocol (‘declined’ or ‘unable to be reached’), was that they benefitted from the experience. While some participants reported quitting or cutting down on their smoking, others reported an increased desire to quit, more awareness of their smoking behavior, or some other form of incremental progress in smoking cessation:

“It was helpful. It definitely was. When I do wanna smoke a cigarette, that (counseling advice) always plays in my ear. So I’ll never forget that every time I pick up a cigarette. So that’s making it better for me.” [ID #49]

Another finding was that several participants who completed the QL program, or were no longer receiving calls because they had been categorized as declined or unreachable, desired continued cessation support. One patient had agreed to the eReferral, but was in the hospital for an emergency surgery when the QL called, so she declined to speak with them at that time:

“Yes, I would (like to talk to the QL), because it was just a misunderstanding and the wrong moment. That’s all it was.” [ID #19]

Another patient had completed her 5 counseling sessions with the QL, but had not completely quit smoking yet:

“Yeah. I’d still like to talk to them. That way we can still set goals and have the motivation to keep going.” [ID #54]

With regard to the larger, primary care context of smoking cessation, the overwhelming majority of patients reported wanting providers’ continued offers of assistance and support with smoking cessation. When asked what role they wanted their primary care providers to play in their smoking cessation, participants expressed the desire for ongoing assistance and encouragement:

“‘If this don’t work, let’s go to the next level,’ you know. In other words, let’s not give up on me. You know, Come on - you keep trying until we find the right thing for you.’ That’s what I want.” [ID #14]

“Just check up to see how I’m doing. Am I still smoking? Am I not smoking? ‘How are you doing with your smoking?’ or ‘If you are still smoking, is there something else that we can do to help you stop smoking?’ Those type of questions.” [ID#42]

## Discussion

Electronic referrals to QLs are recommended by the Community Preventive Services Task Force as an effective intervention for increasing tobacco cessation among patients interested in quitting [23], however, rates of QL program completion are poor [15, 17, 18]. This study expands our understanding of why so many smokers, having accepted a referral during their primary care office visit, later disengage with the QL. Those designated by the QL as having ‘declined’ or been ‘unreachable’ communicated a wide variety of reasons for their non-completion of the 5-session counseling protocol. Interpretation of these categories should be done with care, as they encompass both intentional and unintentional disengagement, and are often based on changing circumstances that affect participants’ ability to engage or their readiness to quit smoking.

Participants reported a variety of stressors that could lead to QL program non-completion. Caregiving for an elderly parent or child - in addition to other responsibilities - was cited as a major stressor, particularly for women. Since 53–68% of caregivers are women, and female caregivers spend more time providing care than male caregivers [24], addressing this barrier could have a substantial impact. Many participants in our sample cited stressors related to lower SES - including poor and unsustainable living conditions, crime and violence, and long and/or unpredictable work hours - as reasons for having to discontinue participation in the QL program. Other studies have also found that stressors related to lower SES are a barrier to use of QL counseling [25, 26]. Smoking rates are highest among lower SES individuals [2, 27], and the use of QLs has the potential to increase access to treatment for lower SES smokers [28, 29]. However, it has also been reported that there remain significant SES disparities in treatment outcomes [30], and the reasons cited above could be mediating variables. Several recent studies have highlighted the importance of providing additional support to manage the stressors (e.g. lack of food, shelter, safety) that can impede smokers’ ability to engage in programs and make/sustain behavior change [31–33]. Integrating support for smoking cessation with referrals to community-based resources to address unmet basic needs has shown promise in several studies [31, 33, 34]. In one such study, smoking cessation rate did not differ by a request for a community referral, but the small number of participants who did use their referral (*n* = 24) were more likely to quit than those who did not (43.6%vs 15.3%; *P* < .001) [35].

Misconceptions about or discomfort with the telephonic format of the QL program was another reason for QL disengagement. Although in this study the MAs who referred patients to the QL first assessed readiness to quit smoking and interest in participating in the QL, several patients expressed that they did not have a clear understanding of what would happen next, or what QL counseling consisted of. More specific yet brief messaging about what to expect from QL counseling could help reduce such misunderstandings, and still remain realistically integrated in the clinical workflow. In addition, offering additional options for cessation support, such as text- or web-based programs, is increasingly shown to be efficacious [36–38]. Another way to reduce discomfort and enhance engagement with QL services could be to incorporate culturally specific interventions for smoking cessation [39, 40]. Cultural adaptations to interventions can be effective in facilitating behavior change, as different ethnic/minority groups can have differing cultural norms and values with regard to smoking, and experience unique barriers to cessation [41, 42]. Providing access to a video-based culturally specific intervention in addition to the QL is one option currently being assessed [40]. In addition, many state QLs are offering services in non-English languages, and expanding services tailored to variety of priority groups, including African Americans, individuals of low SES, and the LGBT community [43].

Problems with cell phones or phone service, including the inability to immediately replace lost, stolen or broken phones, was also reported as a barrier to QL engagement. Among low income smokers, cell phones are often the only form of telephone service [44, 45]. Gonzales et al. found that temporary phone disconnection among low-income patients was frequent, and often caused disruption in access to healthcare [46]. Others have suggested that limited minutes among low income cell phone users could impose a burden on calling plans and therefore deter use of the QL [20, 45], although this was not reported in this study. Making the QL identifiable on caller ID could help improve patient engagement.

The QL categories of ‘program complete’ and ‘quit status’ are often used as indicators of the degree of QL engagement and the success of a smoking cessation program. Our findings suggest that these categories do not tell the whole story. Many participants who did not complete the program found great value in their engagement with the QL, and accomplished goals such as quitting or cutting down on their use of tobacco products. Since smoking cessation is a process that frequently involves several quit attempts, as well as a behavior change that needs to be sustained over time, the QL can potentially play an important role regardless of where a person may be along the smoking cessation trajectory. We found that some individuals who were no longer engaged with the QL – because they had been classified as program complete, unreachable, or declined – expressed that they would value ongoing or future support from the QL. Other studies have found that many relapsed smokers are interested in recycling back in into treatment [47, 48], and that interventions to encourage past QL participants to recycle into services and reinitiate QL-assisted quit attempts are effective [49, 50].

Finally, we found that study participants want ongoing communication with their primary care providers about smoking and smoking cessation. Regardless of their quit status, patients appreciate their providers checking in with them, offering encouragement, and working with them to problem-solve around cessation strategies. Closed-loop EHR referral systems that include delivery of treatment information from the QL back to the provider may help facilitate ongoing patient-provider communication [14].

### Strengths and limitations

Our findings are specific to barriers driven by low socio-economic status, which is reflective of this sample drawn from a health system serving patients who are predominantly low-income. Other barriers may be present in samples with higher socio-economic status. However, the robust sampling strategy to represent perspectives from different types of experiences with the QL and the careful analyses of the sample of 55 individuals are strengths. Efforts were made by the interviewers to limit social desirability bias during the interview process in order to elicit honest responses from participants regarding their experiences with the primary care practices, the QL, and with quitting. However, some participants still may have provided answers that were not completely true to their experiences, opinions, or behaviors. In addition, we engaged people willing to talk with us on the phone, those who did not engage may include individuals who did not want to use up phone minutes with QL counseling.

## Conclusions

This study expands our understanding of why people who use tobacco disengage from a multi-session QL counseling program. QL categories ‘declined’, ‘unreachable’, and ‘program complete’ do not tell the whole story. Participants intentionally and unintentionally disengage for a wide variety of reasons, some of which can be addressed by integrating other approaches into the eReferral to QL process.

## Supplementary information

**Additional file 1.** Qualitative Interview guide. The semi-structured interview guide used to interview study participants who agreed to be referred to the quitline.

## Data Availability

The data generated and analyzed during this study are not publicly available due to data security agreements with MetroHealth, but data are available from the corresponding author upon reasonable request with permission from MetroHealth.
