# Supplementary material for: Quitting the quitline: a qualitative study of patient experience of electronic referrals to quitlines
Source: BMC Public Health. 2020 Jul 9;20:1080. doi: 10.1186/s12889-020-09185-4 (PMC7350715; doi:10.1186/s12889-020-09185-4)
Supplement: Supplementary file 1 — Additional file 1. Qualitative Interview guide. The semi-structured interview guide used to interview study participants who agreed to be referred to the quitline. [file 12889_2020_9185_MOESM1_ESM.docx]

**PATIENT INTERVIEW GUIDE**

- **TURN AUDIO RECORDER ON -**

You might remember about [NUMBER] weeks ago on a [DAY OF THE WEEK] you visited [HEALTH CENTER] to see Dr. [NAME]; it was [MONTH, DATE]. I would like to talk to you about the person who took you to the exam room. We’re going to call that person a medical technical assistant – or the MTA - for the purpose of this interview. The MTA talked to you about your smoking.

**General questions:**

1. Can you recall that visit? Can you tell me about what the MTA said about tobacco?

- What questions did the MTA ask about smoking?
- What did the MTA tell you about smoking?
- What services, if any, did the MTA tell you about?

1. How did you respond to the MTA?

- Can you walk me through what happened?
- How did you respond when the MTA asked about your smoking? Advised you to quit? Asked if you were interested in resources?

1. Thinking about this conversation with the MTA, how did it make you feel? What were you thinking at the time?
2. Was it a good time to bring up smoking for you? Why or why not?
3. What did you find most helpful about your discussion with the MTA? Why is that?
4. Was there anything that was *not* helpful? If so, how was it not helpful?
5. Do you remember accepting a referral to the Ohio quitline?

- Did the MTA or your doctor refer you?
- Did you agree to be connected to the Quitline?
- Did you feel ready to quit when you accepted the referral?
- Did you feel pressured to accept the referral?

1. Can you tell me about what your thoughts were then?

- Why did you want to be connected to the Quitline?
- What were you expecting would happen next?
- Were you expecting counseling over the phone or in person? What makes [phone or in-person] counseling a better option for you?
- How were you hoping the Quitline could help you quit?

1. Did the MTA review the summary report of your doctor’s visit with you at the end of your appointment?

- Did he or she review the phone number for the quitline with you?
- Did the he or she confirm your telephone number with you during your visit?

1. Thinking about your future visits, how do you think the MTA could make this experience better for you?
2. Do you feel like you are treated with respect when discussing smoking with the MTA? Tell me more about that. What made you feel that way?

**Previous quit attempt questions:**

I’d like to switch gears and talk to you about your past history of smoking.

1. Have you tried to quit using tobacco before?

- If yes, tell me about your most recent quit attempt.
- When was this?
- How did it go?
- Did you experience any obstacles that made it difficult to quit smoking? If so, what were they?

1. How long did you quit for? What happened to make you slip and start smoking again?

- What is the main reason why you are continuing to smoke currently?

**Determine contact/extent of contact with Quitline**

1. Has the Quitline contacted you? Tell me about that.

- Do you answer 1-800 calls? Did you recognize the 1-800 number when the quitline called?
- How long ago was that?
- Did they leave a message? How many?
- Did you listen to the message?
- Was it an automated message or a real person?

1. Have you spoken to a person from the Quitline? If so, when you spoke to this person, what did you talk about?

- What did the counselor ask you?
- What advice did the counselor give you?
- How many different times did you speak with someone from the Quitline?

TRY TO DETERMINE REFERRAL CATEGORY BEFORE PROCEEDING

**Questions for those who ACCEPTED referral:**

1. Now I’d like to talk specifically about your conversation with the Quitline counselor. Can you tell me about that?

- What made you want to talk to the Quitline?
- How did your conversation go? How did it make you feel?
- What did you talk about?
- What did the counselor ask/tell you?
- What was most useful to you about the counseling?
- Was there anything that was not useful to you?
- How many different times did you speak with someone from the Quitline? Tell me more about that.

**Questions for those who DECLINED the referral:**

1. Now I’d like you to think specifically about your decision **not** to be connected to a coach or counselor. Can you tell me about that?

- What were you thinking at the time?
- Why did you decide to turn down the program if you still want to quit?
- Was there anything that you did not like about the quitline? Was there something about the interaction that made you decline?
- How was your experience different from what you expected?
- Is there anything that would make you more willing to enroll in the future? Tell me more about that.
- What are your thoughts about quitting tobacco use in the future?
- What would be most useful to you to help you the next time you want to quit?

**Questions for those who accepted the referral but were UNREACHABLE:**

1. Now I’d like to talk specifically about your decision to be connected to a coach or counselor to help you quit. Can you tell me about that? What were you thinking at the time?

- What happened when the Quitline called/left a message? How did you respond?
- What were you thinking and feeling when the Quitline called/left a message?
- Did the quitline try to contact you in any other way? Letter in the mail? If so, what was in the content of the letter? How did you respond?

1. We’re really interested to know why people have not made the connection to the quitline for research purposes. There really is no right or wrong answer. Can you tell us more?

- During your discussion about smoking with the MTA, you indicated that you were ready to quit and wanted to be referred for counseling services. What made you change your mind?
- Would you respond in the same way if they called you tomorrow? Why or why not?
- Do you still want the quitline to call? Why or why not?
- Do you plan on calling the quitline?
- Are there any other reasons why you haven't called them back (besides being busy)? (ie. bad timing, miscommunication, or not interested)

1. Why do you think they didn’t contact you? (If applicable)

- Is there any reason why the quitline may have trouble contacting you? Have you changed your number recently?

**NRT questions:**

1. Now I’d like to ask you a couple questions about using medication to help you quit. What do you think about using nicotine replacement therapy such as the patch, gum, or lozenge?

- Has anyone prescribed that for you? Who?
- Have you ever purchased or borrowed any of these products?
- What has been your experience in using this medication?
- What have you heard about others’ experiences using this medication?
- (If applicable) What are your concerns about using this medication?
- What would make you willing to try medication for smoking cessation?

1. Have you ever heard of Chantix or Wellbutrin?

- What are your thoughts about using these medications to help you quit smoking?
- Have you had any experience using them? If so, tell me about your experience.
- What did you like about taking this medication?
- What didn’t you like about taking the medication?
- Have you heard stories or seen commercials about the medication? If so, how did it make you feel?

**Final general questions:**

1. Now I’d like you to think about your healthcare team as a whole. This would include all the people you interact with when you go to an appointment – the MTA, the nurse, the physician. What role do you want your healthcare team to play in terms of your smoking?

- What would you like them to say to you or do for you?
- What would you not like them to say to you or do for you?

1. How else can your healthcare team help you quit?

- What other services or information can help you quit?
- For example, would you be interested in group classes? Why or why not? If so, how often would you like to take these classes?

1. Is there anything else you would like to share with us about your experience with the quitline or your healthcare team as it relates to helping you quit tobacco?

**Final message about contacting the quitline:**

You can always call the quitline if you are still interested. They can be reached at 1-800 QUIT-NOW. (if necessary)

**Conclusion:**

This now concludes our interview. Thank you for taking the time to participate in our research study.

**PATIENT INCENTIVES**

- **TURN AUDIO RECORDER OFF -**

You are eligible to receive a $25 gift card to Target or Amazon. Do you have a preference, Target or Amazon?

We can send you the gift card in the mail. What is your mailing address?

[PLEASE, REMEMBER TO CONFIRM ALL MAILING ADDRESS]

Once again, thank you for your participation. You can expect to receive the gift card in up to 3 weeks. If you have any further questions, please feel free to call me back. I can be reached at [PHONE NUMBER].

**END CONTACT**
